# Role of Cancer History in Cardiovascular Mortality Among Different Age-group Patients With Differentiated Thyroid Cancer

**DOI:** 10.1210/jendso/bvae213

**Published:** 2024-11-26

**Authors:** Hongrui Qiu, Wenyi Zhou, Qizhi Huang, Hongwei Lin, Yubo Zhou, Chaodong Wu, Yijie Huang, Jinhang Leng

**Affiliations:** Department of Thoracic Surgery, Guangdong Cardiovascular Institute, Guangdong Provincial People’s Hospital (Guangdong Academy of Medical Sciences), Southern Medical University, Guangzhou, 510080 Guangdong, China; Department of Immunology, Zhongshan School of Medicine, Sun Yat-sen University, Guangzhou, 510080 Guangdong, China; Guangdong Provincial Key Laboratory of Malignant Tumor Epigenetics and Gene Regulation, Medical Research Center, Sun Yat-Sen Memorial Hospital, Sun Yat-Sen University, Guangzhou, 510120 Guangdong, China; Yat-Sen Breast Tumor Hospital, Sun Yat-Sen Memorial Hospital, Sun Yat-Sen University, Guangzhou, 510120 Guangdong, China; Department of Thoracic Surgery, Guangdong Cardiovascular Institute, Guangdong Provincial People’s Hospital (Guangdong Academy of Medical Sciences), Southern Medical University, Guangzhou, 510080 Guangdong, China; Guangdong Cardiovascular Institute, Guangdong Provincial People’s Hospital, Guangdong Academy of Medical Sciences, Guangzhou, 510080 Guangdong, China; Department of Thoracic Surgery, Guangdong Cardiovascular Institute, Guangdong Provincial People’s Hospital (Guangdong Academy of Medical Sciences), Southern Medical University, Guangzhou, 510080 Guangdong, China; The First School of Clinical Medicine, Guangdong Medical University, Zhanjiang, 524023 Guangdong, China; School of Chinese Medicine, Faculty of Medicine, Macau University of Science and Technology, 999078 Macao, China; The First Clinical Medical School, Southern Medical University, Guangzhou, 510515 Guangdong, China; Department of General Surgery, Guangdong Provincial People’s Hospital (Guangdong Academy of Medical Sciences), Southern Medical University, Guangzhou, 510080 Guangdong, China; Department of Thoracic Surgery, Guangdong Cardiovascular Institute, Guangdong Provincial People’s Hospital (Guangdong Academy of Medical Sciences), Southern Medical University, Guangzhou, 510080 Guangdong, China

**Keywords:** differentiated thyroid cancer, cardiovascular disease, mortality risk, second primary neoplasm, cancer treatment, SEER program

## Abstract

**Background:**

Cardiovascular disease (CVD) is the leading cause of noncancer-related mortality among differentiated thyroid cancer (DTC) survivors, which accounts for a large portion of subsequent primary malignancies in childhood cancer survivors. This study aims to assess the risk of cardiovascular mortality among DTC as a second primary malignancy (DTC-2) patients compared with DTC as a first primary malignancy (DTC-1) and the general population.

**Methods:**

Using the Surveillance, Epidemiology, and End Results database, we conducted a population-based cohort study including 159 395 DTC-1 and 20 010 DTC-2 patients diagnosed older than 30 between 1975 and 2020 and the corresponding US population (71 214 642 person-years; 41 420 893 cardiovascular deaths). Compared with general-population and DTC-1 patients, we calculated incidence rate ratios (IRRs) of cardiovascular deaths among DTC-2 patients using Poisson regression. To adjust for unmeasured confounders, we performed a nested, case-crossover analysis among DTC-2 patients who died from CVD.

**Results:**

Although DTC-2 patients had a comparable risk compared with the population (IRR 1.01) and a mildly increased risk of cardiovascular mortality compared with DTC-1 patients (IRR 1.26), the association was pronounced among individuals aged 30 to 74 years, especially 30 to 44 years (DTC-2 vs population: IRR 8.89; DTC-2 vs DTC-1: IRR 3.00). The risk elevation was greatest within the first month after diagnosis, compared with the population. The case-crossover analysis confirmed these results.

**Conclusion:**

DTC-2 patients are at increased risk of cardiovascular mortality. Clinicians should carefully monitor CVD and manage other CVD-related factors, such as exogenous thyroxine and emotional distress, for DTC-2 patients, especially for those under 75 years.

**Novelty and Impact Statements:**

This study is the first comprehensive investigation into the cardiovascular mortality of DTC-2, revealing a higher risk compared to DTC-1 and the general population, especially for cases between 30 and 74 years old. The risk elevation was greatest within the first month after diagnosis. These findings emphasize the restriction of thyroid hormone suppression therapy and reinforce stress management to prevent premature DTC-2 patients from cardiovascular death.

Thyroid cancer is the most prevalent endocrine malignancy, accounting for 3% of all newly diagnosed cancer cases in the United States and ranking as the eighth most common cancer among women [1]. Advances in the detection of thyroid tumors and enhancements in therapeutic approaches have yielded favorable prognoses and low mortality rates for this disease [2]. Nevertheless, causes unrelated to cancer are responsible for 46.4% of deaths in thyroid cancer survivors, with cardiovascular diseases (CVD) emerging as a primary contributor [3-5]. Differentiated thyroid carcinoma (DTC), which includes both papillary and follicular thyroid carcinoma, is the most common subtype of thyroid cancer. Patients with a primary diagnosis of DTC (DTC-1) face an elevated risk for CVD, potentially linked to shared risk factors such as cigarette smoking, alcohol consumption, and obesity [6-8], the adverse effects of cancer treatment [9], and psychological distress [10]. Some studies [11, 12] have indicated an increased risk of cardiovascular mortality in patients with DTC compared to the general population.

With the extension of cancer survivorship, second primary malignancies have become increasingly frequent [13]. Notably, approximately 10% of subsequent primary malignancies in survivors of childhood cancers are thyroid-related [14]. The presence of a previous malignancy further promotes CVD of DTC patients through many mechanisms. First, the influence of chemotherapy and radiotherapy on the risk of CVD in cancer patients has been documented in prior studies [15]. Recommendations for lifetime dose limitations exist for specific cancer treatments, such as radiotherapy and anthracycline-based chemotherapy [16, 17]. Although exceeding the dose limit for a given regimen due to a second malignancy is uncommon, patients with DTC as a secondary malignancy (DTC-2) may have been treated with anthracyclines for their initial malignancy and radioactive iodine treatment for DTC-2, which jointly lead to the occurrence of CVD. Second, to prevent recurrence risk, postoperative thyroid hormone suppression therapy (THST) to decrease TSH levels through administration of excessive exogenous T4 has been a longstanding standard for all patients with DTC for decades [18]. A correlation between lower TSH levels and increased cardiovascular mortality has been reported [12]. Hence, THST for DTC-2 may impose extra cardiotoxicity. Third, the recent occurrence of a previous malignancy, a considerable stressor, is associated with higher cardiovascular mortality [19]. It is plausible to suppose that a DTC-2 diagnosis may further magnify the risk of cardiovascular death.

This risk of cardiovascular mortality is less explored in younger cohorts, although increased premature CVD mortality has been reported in individuals under 40 diagnosed with DTC-1 [20]. A Taiwanese study also observed an intensified association between thyroid cancer and coronary heart disease risk among patients diagnosed with thyroid cancer before 65. In addition, previous research has highlighted a rise in cardiovascular mortality in a relatively short time following a DTC-1 diagnosis [19, 21]. In this study, we aim to assess the relative risk of cardiovascular mortality for DTC-2 patients compared to DTC-1 patients and the general population, as well as the variation of the risk across different attained age groups and different periods after diagnosis. Considering the sex differences in cancer prevalence and CVD outcomes, we also conducted a subgroup analysis stratified by sex. To adjust for unmeasured confounders shared by DTC and CVD, we then performed a nested, case-crossover analysis among DTC-2 patients who died from CVD.

## Materials and Methods

### Study Design

**Figure 1.**
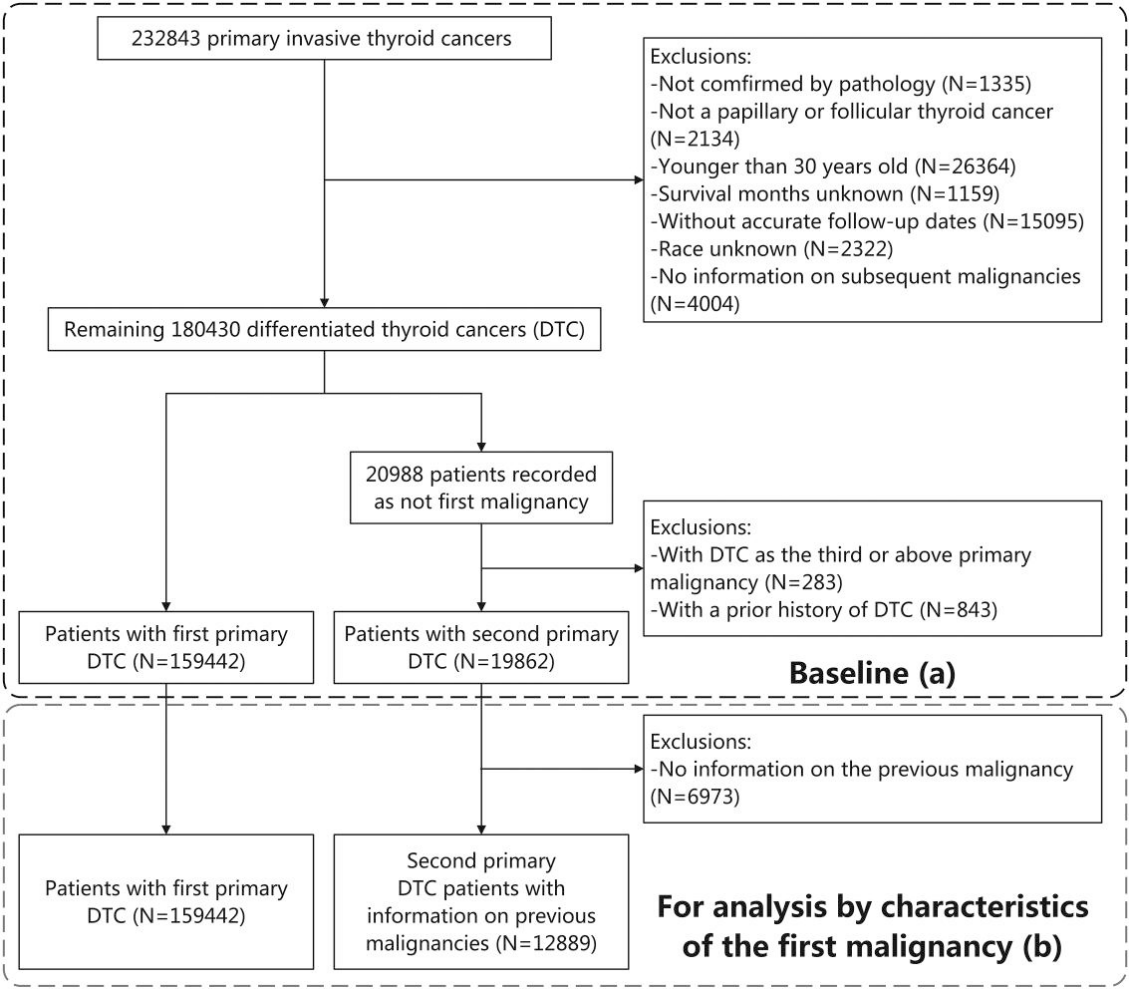
Selection of the baseline cohort (A) and the generation of the cohort for analysis by characteristics of the first malignancy (B). Abbreviatiins: DTC, differentiated thyroid cancer.

A previous study demonstrated that the age group of older than 30 years had a much higher age-standardized cardiovascular mortality rate compared to the age range of 0 to 30 years [22]. Thus, we focus on people over 30 years old who are more exposed to CVD. Based on the Surveillance, Epidemiology, and End Results (SEER) database, we conducted a population-based cohort study of patients aged more than 30 years with primary DTC diagnosed from January 1, 1975, to December 31, 2020 (between 1975 and 1991 for SEER-9; between 1992 and 1999 for SEER-13; between 2000 and 2020 for SEER-18). We identified 232 843 patients with primary thyroid cancer. We further excluded patients who were not confirmed by pathological diagnosis (n = 1335), did not have papillary or follicular thyroid cancer (n = 2134), were younger than 30 years at diagnosis (n = 26 364), and whose survival months or race was unknown (n = 1159; n = 2322). Patients were followed from DTC diagnosis until death, the occurrence of a subsequent malignancy, or December 31, 2022, whichever occurred first; thus patients without information on subsequent malignancy were also excluded (n = 4004). We excluded patients without accurate follow-up dates (n = 15,095, Fig. 1). DTC-2 accounted for 6.4% of new cases between 1975 and 1989, and this proportion increased to 13.0% between 2017 and 2020 (Supplementary Fig. S1, [23]).

Using the National Cancer Institute, DCCPS, Surveillance Research Program, we also included 7 121 464 230 person-years from the total US population 1975 to 2020 [24].

### Ascertainment of DTC-2

There were 21 035 individuals not diagnosed as their first malignancy among the remaining 180 430 DTC patients. By connecting the prior malignancy records, we excluded DTC-2 patients with a previous history of DTC (n = 742) such as contralateral DTC, due to the complexity of ascertaining cause of death. Finally, we included 179 405 patients with primary DTC (20 010 DTC-2 and 159 395 DTC-1). For analysis based on the characteristics of the first malignancy, patients with no information on prior malignancies were further excluded (n = 7,121, Fig. 1).

### Ascertainment of Cardiovascular Deaths and Follow-up

We identified deaths by CVD by the International Classification of Diseases codes (Supplementary Table S1, [23, 25]). Subgroups of cardiovascular deaths were analyzed, including heart disease, cerebrovascular disease, and other CVD. Given that the risk of cardiovascular death is closely related to age, which increases along with follow-up [26], we applied attained age into our analyses instead of age at diagnosis for better confounding adjustment.

### Statistical Analysis

We applied a Poisson regression model to estimate the incidence rate ratios (IRRs) and 95% confidence intervals (CIs) of cardiovascular deaths among DTC-2 patients, compared with the general population and DTC-1 patients, respectively. To address concerns regarding the different risks of subsequent malignancy development between DTC-1 and DTC-2 patients, we additionally conducted an analysis without censoring follow-up at the occurrence of subsequent malignancy. Furthermore, additional analyses based on age at DTC diagnosis and sex were performed, considering their clinical significance. Subsequent analyses were conducted in the attained age group of 30 to 74, as the increased risk was most obvious in DTC-2 patients aged within this range.

For comparisons between DTC-2 patients and DTC-1 patients or the general population, the analyses initially controlled for demographic characteristics (model A). Additional adjustments were made for tumor features (model B) and treatment modalities (model C, ie, full model) when comparing DTC-2 patients with DTC-1 patients. Covariates used in the models are summarized in Supplementary Table S2 [23]. In subgroup analyses, only model C was applied. To further evaluate the influence of the first malignancy on cardiovascular mortality of DTC-2, we performed subgroup analysis by characteristics and treatment modality of the prior cancer. The sites as well as the corresponding codes in the International Classification of Diseases, Tenth Revision of the smoking-related cancers and the thoracic cancers are recorded in Supplementary Table S3 [23, 27, 28].

To balance shared risk factors between DTC and CVD (eg, smoking, obesity, and excessive drinking) and comorbidities (eg, dyslipidemia and previous history of CVD), a self-matched, nested case-control analysis [29] was conducted among DTC-2 patients who died of CVD. Following the method described by Fang et al [19], we compared the incidence of DTC-2 diagnosis during the first month preceding cardiovascular death (hazard period) with that of 17 1-month periods before the hazard period (control periods). The case-crossover analysis enabled patients to act as their own controls to minimize confounding by individual risk factors. We used conditional logistic regression to calculate the odds ratios (ORs) of the hazard period compared to the control period.

All statistical analyses were performed using R (version 3.6.3; The R Foundation). Statistical significance was defined as *P* < .05. The research obtained approval from the Ethics Committee at Guangdong Provincial People's Hospital, ensuring adherence to ethical guidelines and protocols.

## Results

### Demographic and Clinical Characteristics

Our analysis revealed that DTC-2 patients were generally older, predominantly White, and more frequently diagnosed after 2007, in comparison to the general population and DTC-1 patients. There was a lower incidence of radiotherapy treatment among DTC-2 tumors when compared to DTC-1. Additionally, a smaller proportion of DTC-2 patients had a follow-up duration of 10 years postdiagnosis (Supplementary Table S2, [23]). The distribution of the prior cancer sites are shown in Supplementary Fig. S2 [23]. The breast, skin, and prostate were the most common sites of the first malignancy.

### Cardiovascular Mortality Risk by Attained Age

Over a median follow-up period of 6.7 years (interquartile range, 2.8-11.7 years), we recorded 737 cardiovascular deaths among DTC-2 patients (mortality rate: 0.70 per 100 person-years) and 3612 among DTC-1 patients (mortality rate: 0.29 per 100 person-years). The median follow-up for DTC-1 was 6.9 years (interquartile range, 3.0-12.1 years). By 20 years postdiagnosis, the cumulative thyroid cancer-specific mortality for DTC-2 was slightly elevated than that for DTC-1 (5.82% vs 4.97%; Supplementary Fig. S3A, [23]), while the cumulative cardiovascular mortality for DTC-2 was notably higher than that for DTC-1 patients whether considering competing risk (9.46% vs 4.94%; Supplementary Fig. S3B, [23]) or not (4.03% vs 2.37%; Supplementary Fig. S4, [23]). The general population experienced 41 420 893 cardiovascular deaths (mortality rate: 0.58 per 100 person-years).

Upon adjusting for demographic variables, the cardiovascular mortality risk for DTC-2 patients was comparable to that of the general population (IRR 1.01, 95% CI 0.94-1.26; Table 1). However, an age-stratified analysis showed a significantly increased risk for younger DTC-2 patients aged 30 to 44 years (IRR 8.89, 95% CI 3.02-26.12), with the risk diminishing after the age of 75 (IRR 0.36, 95% CI 0.18-0.73; Table 1). This age-dependent risk pattern was also evident when contrasting DTC-2 with DTC-1 patients, irrespective of tumor characteristics and treatment modalities, with DTC-2 consistently at higher risk (IRR 1.26, 95% CI 1.17-1.37; Table 1). The analysis without censoring follow-up at subsequent malignancy onset corroborated these findings (Supplementary Table S4, [23]). Additionally, age-based grouping of DTC-1 and DTC-2 patients at the time of DTC diagnosis showed similar trends to the primary analyses focused on attained age (Supplementary Table S5, [23]).

**Table 1.** IRRs of cardiovascular deaths among patients with the primary DTC-2 developed from nonmammary malignancy, compared with population and patients with DTC-1: a population-based study in the United States, 1975-2020

	References		DTC-2 n (MR)	DTC-2 vs Population	DTC-2 vs DTC-1		
	Population n (MR)	DTC-1 n (MR)		IRR (95% CI)*^a^*	IRR (95% CI)*^b^*	IRR (95% CI)*^c^*	IRR (95% CI)*^d^*
Overall	41 420 893 (0.58)	3612 (0.29)	737 (0.70)	1.01 (0.94-1.26)	1.27 (1.18-1.38)	1.27 (1.18-1.38)	1.26 (1.17-1.37)
By attained age, yr							
30-44	812 453 (0.03)	62 (0.06)	8 (0.20)	8.89 (3.02-26.12)	2.86 (1.33-6.16)	3.29 (1.65-6.54)	3.00 (1.50-6.02)
45-54	2 012 101 (0.13)	253 (0.09)	27 (0.20)	2.39 (1.21-3.65)	1.62 (1.04-2.51)	1.65 (1.06-2.56)	1.85 (1.22-2.79)
55-64	4 467 967 (0.35)	481 (0.12)	73 (0.26)	1.43 (1.03-1.97)	1.44 (1.11-1.86)	1.45 (1.12-1.89)	1.36 (1.04-1.79)
65-74	8 079 918 (0.88)	770 (0.23)	155 (0.45)	0.78 (0.54-1.34)	1.28 (1.08-1.51)	1.29 (1.09-1.53)	1.28 (1.08-1.52)
≥75	26 048 454 (3.67)	2046 (0.91)	474 (1.37)	0.36 (0.18-0.73)	1.19 (1.09-1.30)	1.19 (1.09-1.30)	1.19 (1.08-1.30)
*P* for interaction*^e^*				<.001	<.001	<.001	<.001

Abbreviations: CI confidence interval; DTC-1, differentiated thyroid cancer as a first malignancy; DTC-2, differentiated thyroid cancer as a second malignancy; IRR, incidence rate ratio, MR, mortality rate per 100 person-years; n, number of deaths.

^*a*^IRRs were adjusted for sex (female or male), attained age (30-44, 45-54, 55-64, 65-74, or ≥75 years), race (White, Black, or other), and calendar year at follow-up (1975-1989, 1990-1993, 1994-1997, 1998-2001, 2002-2006, 2007-2011, 2012-2016, or 2017-2020). Patients who were not White/Black were grouped at the national level when comparing DTC-2 with the population.

^*b*^IRRs were additionally adjusted for time since diagnosis (0 to <1 month, 1 to <6 months, 6 to <12 months, 1 to <2 years, 2 to <5 years, 5 to <10 years, or ≥10 years).

^*c*^IRRs were additionally adjusted for tumor stage (localized, regional, distant, or unknown), histology (follicular or papillary), and grade (well differentiation, moderate differentiation, poor differentiation, undifferentiation, or unknown).

^*d*^IRRs were additionally adjusted for surgery (yes or no/unknown), radiotherapy (yes or no/unknown), and chemotherapy (yes or no/unknown).

^*e*^We added an interaction term between DTC-2 and attained age (30-44, 45-54, 55-64, 65-74, or ≥75 years) and reported the significance level of the term as *P* for interaction.

The interaction with attained age prompted further stratification and analysis. The subgroup analysis shows the age-dependent risk pattern still exists in both sex subgroups, although the disparity of that is more obvious in female patients (Supplementary Table S6, [23]). For patients aged 30 to 74 years, more notable associations with cardiovascular mortality from heart disease, cerebrovascular conditions, and other cardiovascular causes were observed when comparing DTC-2 patients to both the general population and DTC-1 patients (Table 2).

**Table 2.** IRRs of type-specific cardiovascular deaths among patients with the primary DTC-2 developed from nonthyroidal malignancy, compared with the population and patients with primary DTC-1: a population-based study in the United States, 1975-2020

	Population, n (MR)	DTC-1, n (MR)	DTC-2, n (MR)	DTC-2 vs Population IRR (95% CI)*^a^*	DTC-2 vs DTC-1 IRR (95% CI)*^b^*
Overall cardiovascular deaths					
By attained age					
30-74 years	15 372 470 (0.24)	1566 (0.14)	263 (0.33)	1.96 (1.16-3.31)	1.73 (1.51-1.97)
≥75 years	26 048 423 (3.67)	2046 (0.91)	474 (1.37)	0.36 (0.18-0.73)	1.19 (1.08-1.30)
Due to disease of heart					
By attained age					
30-74 years	12 436 881 (0.19)	1175 (0.11)	208 (0.26)	2.00 (1.17-3.40)	1.77 (1.52-2.07)
≥75 years	19 414 666 (2.74)	1462 (0.67)	351 (1.04)	0.36 (0.18-0.73)	1.24 (1.11-1.38)
Due to cerebrovascular disease					
By attained age					
30-74 years	2 078 910 (0.03)	257 (0.02)	37 (0.05)	1.81 (1.02-3.22)	1.84 (1.29-2.64)
≥75 years	4 884 966 (0.69)	430 (0.21)	86 (0.27)	0.36 (0.17-0.76)	1.11 (0.88-1.41)
Due to other cardiovascular diseases					
By attained age					
30-74 years	856 679 (0.01)	134 (0.01)	18 (0.02)	2.01 (0.99-4.07)	1.41 (0.82-2.43)
≥75 years	1 748 791 (0.25)	154 (0.08)	37 (0.12)	0.40 (0.18-0.88)	1.37 (0.95-1.99)

Abbreviations: CI, confidence interval; DTC-1, differentiated thyroid cancer as a first malignancy; DTC-2, differentiated thyroid cancer as a second malignancy; n, number of deaths; OR, odds ratio.

^*a*^IRRs were adjusted for sex (female or male), attained age (30-44, 45-54, 55-64, 65-74, or ≥75 years), race (White, Black, or other), region of residence (Northeast, Midwest, South, or West) and calendar year at follow-up (1975-1989, 1990-1993, 1994-1997, 1998-2001, 2002-1006, 2007-2011, 2012-2016, or 2017-2020). Patients who were not White/Black were grouped at the national level when comparing DTC-2 with the population.

^*b*^IRRs were additionally adjusted for time since diagnosis (0 to <1 month, 1 to <6 months, 6 to <12 months, 1 to <2 years, 2 to <5 years, 5 to <10 years, and 10 years), tumor stage (localized, regional, distant or unknown), histology (follicular or papillary), grade (well differentiation, moderate differentiation, poor differentiation, undifferentiation or unknown), surgery (yes or no/unknown), radiotherapy (yes or no/unknown) and chemotherapy (yes or no/unknown).

### Cardiovascular Mortality Risk by Time Since Diagnosis

**Figure 2.**
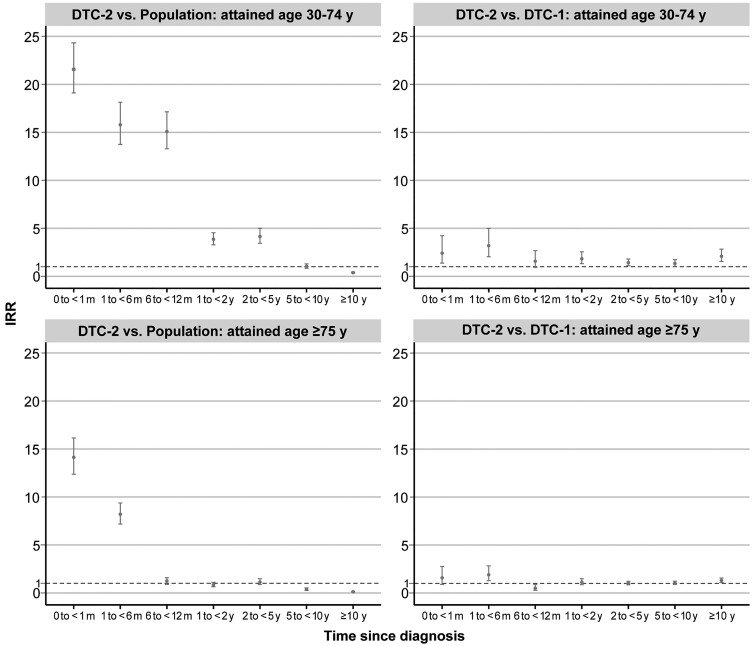
IRRs of cardiovascular deaths among patients with the primary DTC-2 developed from nonthyroidal malignancy by time since cancer diagnosis, compared with the US population and patients with primary DTC-1: a population-based study in the United States, 1975-2020. DTC-2 vs population: the general population served as a constant reference to be compared with different periods after cancer diagnosis in patients with DTC-2. IRRs were adjusted for sex (female or male), attained age (30-44, 45-54, 55-64, 65-74, or ≥75 years), race (White, Black, or other), and calendar year at follow-up (1975-1989, 1990-1993, 1994-1997, 1998-2001, 2002-2006, 2007-2011, 2012-2016, or 2017-2020). Patients who were not White/Black were grouped at the national level when comparing DTC-2 with the population. DTC-2 vs DTC-1: IRRs were additionally adjusted for time since diagnosis (0 to <1 month, 1 to <6 months, 6 to <12 months, 1 to <2 years, 2 to <5 years, 5 to <10 years, and ≥10 years), tumor stage (localized, regional, distant, or unknown), histology (follicular or papillary), grade (well differentiation, moderate differentiation, poor differentiation, undifferentiation, or unknown), surgery (no surgery, yes surgery, or unknown), radiotherapy (yes or no/unknown), and chemotherapy (yes or no/unknown). Abbreviations: DTC-1, differentiated thyroid cancer as a first malignancy; DTC-2, differentiated thyroid cancer as a second malignancy; IRR, incidence rate ratio; m, month; y, years.

In the early postdiagnosis period, DTC-2 patients aged 30 to 74 years exhibited a markedly increased cardiovascular mortality compared with the general population, particularly within the first month (IRR 21.56, 95% CI 19.11-24.32; Fig. 2 and Supplementary Table S7, [23]). When compared with the general population and DTC-1 patients, this elevated risk persisted up to 5 years following the cancer diagnosis. For those over 75 years of age, an increased risk was seen only within the initial month (IRR 14.13, 95% CI 12.37-16.14) and the subsequent 1 to 6 months (IRR 8.20, 95% CI 7.18-9.37) following diagnosis, relative to the general population. A comparable temporal risk pattern was noted in comparisons between DTC-2 and DTC-1 patients.

The case-crossover analysis further highlighted the significant rise in cardiovascular mortality risk during the first-month post-DTC-2 diagnosis, which was consistent both before and after the age of 75 years, compared to reference periods (OR 2.00, 95% CI 1.22-3.26; Table 3). The inherent self-matching mechanism of nested case-control studies eliminates the influence of confounding factors such as smoking, obesity, excessive drinking, dyslipidemia, and previous history of CVD.

**Table 3.** Odds ratios of cardiovascular deaths after the diagnosis of primary DTC-2 developed in survivors of nonthyroidal malignancy in case-crossover analysis*^a^*: a population-based study in the United States, 1975-2020

	Cardiovascular deaths, n (%)		Matched cases, n (%)		OR (95% CI)*^b^*
	Control period	Hazard period	Control period	Hazard period	
Overall	94 (81.7)	21 (18.3)	2380 (89.9)	266 (10.1)	2.00 (1.22-3.26)
By attained age					
30-74 years	34 (75.6)	11 (24.4)	1723 (90.8)	174 (9.2)	3.20 (1.59-6.44)
≥75 years	60 (85.7)	10 (14.3)	657 (87.7)	92 (12.3)	1.19 (0.59-2.41)

Abbreviations: CI, confidence interval; DTC-2, differentiated thyroid cancer as a second malignancy; n, number of deaths; OR, odds ratio.

^*a*^This analysis included all second primary differentiated thyroid cancer patients. The hazard period was defined as the 1 month (30 days) preceding cardiovascular death and the control periods as the 17 1-month periods preceding the hazard period.

^*b*^The OR is the odds a case would have fallen within the hazard period in the cardiovascular deaths group vs that in the matched cases group.

### Factors Modifying Cardiovascular Mortality Risk

**Figure 3.**
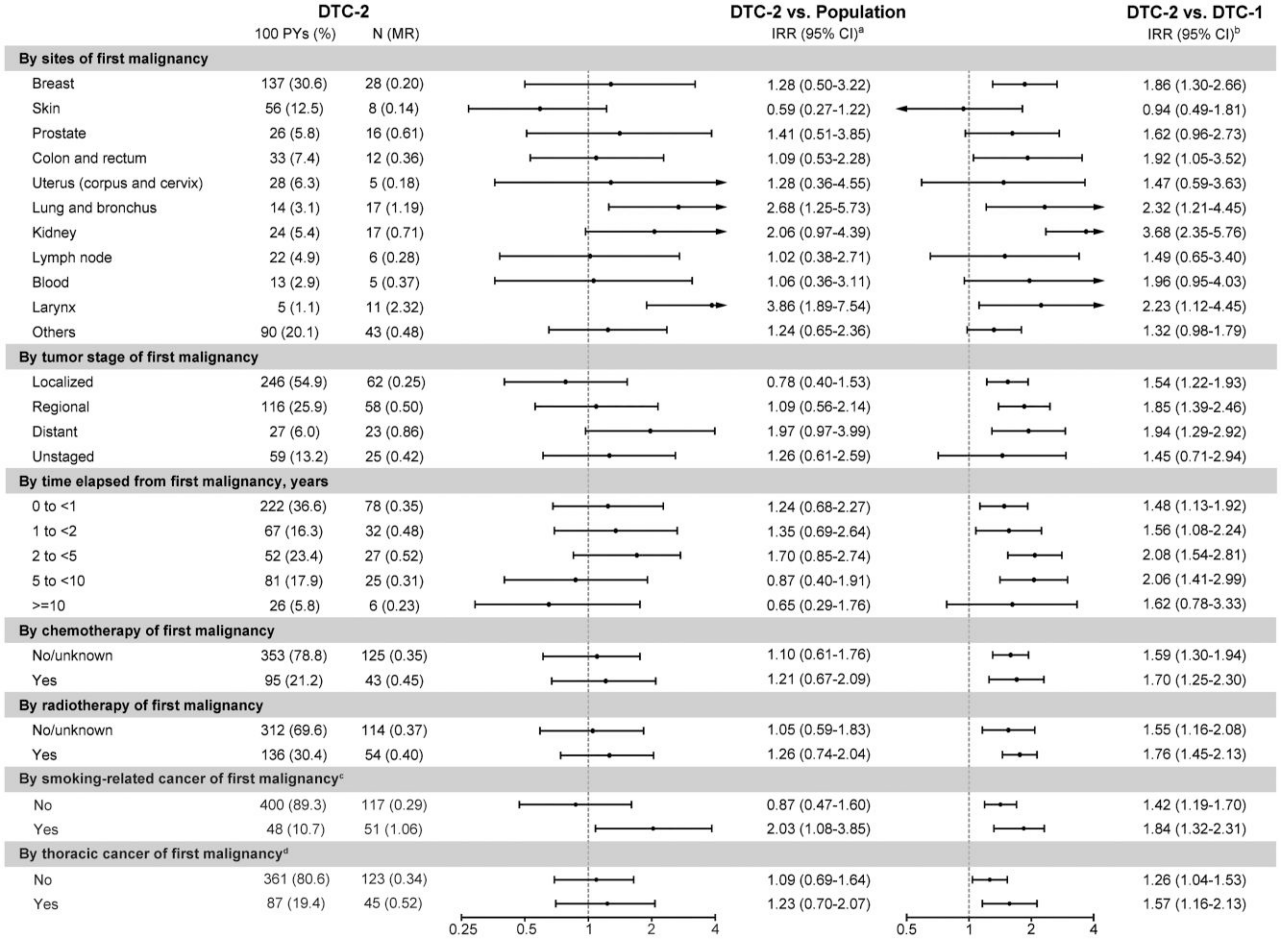
IRRs of cardiovascular deaths at attained age 30 to 74 years among patients with the primary DTC-2 developed from nonthyroidal malignancy by characteristics of the first malignancy, compared with the US population and patients with primary DTC-1: a population-based study in the United States, 1975-2020. ^a^IRRs were adjusted for sex (female or male), attained age (30-44, 45-54, 55-64, 65-74, or ≥ 75 years), race (White, Black, or other), and calendar year at follow-up (1975-1989, 1990-1993, 1994-1997, 1998-2001, 2002-2006, 2007-2011, 2012-2016, or 2017-2020). Patients who were not White/Black were grouped at the national level when comparing DTC-2 with the population. ^b^IRRs were additionally adjusted for time since diagnosis(0 to <1 month, 1 to <6 months, 6 to <12 months, 1 to <2 years, 2 to <5 years, 5 to <10 years, and ≥10 years), tumor stage (localized, regional, distant, or unknown), histology (follicular or papillary), grade (well differentiation, moderate differentiation, poor differentiation, undifferentiation, or unknown), surgery (no surgery, yes surgery, or unknown), radiotherapy (yes or no/unknown), and chemotherapy (yes or no/unknown). ^c^Smoking-related cancers included cancer of lip, oral cavity, or pharynx; esophagus; stomach; colorectum; liver; pancreas; lung, bronchus, or trachea; cervix; bladder; and blood. ^d^The thoracic cancers included cancer of esophagus, lung, bronchus, or trachea; mediastinum and pleura; and breast. Abbreviations: CI: confidence interval; DTC-1, differentiated thyroid cancer as a first malignancy; DTC-2, differentiated thyroid cancer as a second malignancy; IRR, incidence rate ratio; MR, mortality rate per 100 person-years; N, number of deaths.

Subgroup analyses, focusing on patients aged 30 to 74 years due to the nonincreased risk among older patients, demonstrated that DTC-2 patients with prior cancers such as breast, colon and rectum, lung and bronchus, kidney, or larynx had an elevated cardiovascular mortality risk compared with DTC-1 patients (Fig. 3). The risks are higher among those whose initial malignancy was smoking-related cancer or thoracic cancer, at a disseminated stage, diagnosed 2 to 5 years before DTC-2, or those who had undergone chemo-/radiotherapy. The risk heterogeneity was also observed when comparing these DTC-2 patients to the general population though not significant for some subgroups, suggesting an additive effect of previous cancer characteristics and treatment modality on subsequent cardiovascular mortality.

When examining DTC characteristics specifically, the risk of cardiovascular mortality in DTC-2 patients increased significantly for papillary thyroid cancer (IRR 1.46, 95% CI 1.26-1.68), those who were not treated with radiotherapy for their DTC (IRR 1.45, 95% CI 1.23-1.71), and those who received radioactive iodine treatment (IRR 1.32, 95% CI 1.02-1.72), compared with DTC-1 patients. The risk also increased in all subgroups of stages except for distant stage (IRR 0.87, 95% CI 0.46-1.64) (*P* for interaction <.05; Supplementary Table S8, [23]). Though comparable IRRs were observed when comparing these DTC-2 patients to the general population, the interaction test showed a significant *P*-value for tumor stage and radiotherapy, reinforcing the influence of DTC characteristics and therapy on cardiovascular mortality.

## Discussion

In this pioneering study, we explored the cardiovascular mortality risk among cancer survivors with subsequent primary DTC first Our analysis revealed that patients with DTC-2, particularly those aged 30 to 74 years, faced a heightened risk of cardiovascular mortality when compared to both the general population and individuals with DTC-1. Notably, the risk escalation was most pronounced within the first-month postdiagnosis, indicating an acute stress-related impact on cardiovascular health, which was induced by a DTC-2 diagnosis. This observation was robustly corroborated by case-crossover analysis, effectively adjusting for confounders shared between DTC and CVD.

The age-related variation in cardiovascular mortality risk is well documented [30], both in the general population and among thyroid cancer survivors [20]. However, our findings add a unique perspective by highlighting the relative risk intensity in younger DTC-2 patients vs their older counterparts, who, despite a higher absolute risk, demonstrate a decreased relative risk past the age of 75. These findings align with broader cancer research suggesting that younger age at diagnosis is associated with a greater relative risk of cardiovascular mortality [31].

THST, aimed at reducing DTC recurrence risk, has been associated with cardiovascular morbidity through various mechanisms, including atrial fibrillation, decreased arterial elasticity, and altered cardiac function [32-35]. The optimal balance between minimizing potential iatrogenic cardiovascular death and recurrence risk triggered by THST via administration of excessive exogenous T4 has become a challenge for DTC-2 and may differ between different age groups. On the one hand, DTC diagnosed at a higher age has been linked to increased cancer-specific mortality, starting with 40 years old [36]. Recurrence frequencies are highest (40%) for those >60 years; recurrence at other ages ensues in only about 20% of patients [37-39]. Current cancer treatment guidelines, such as those from the American Joint Committee on Cancer, recommend relatively conservative staging criteria and anticancer regimens for younger groups [40]. On the other hand, our results illustrated that younger patients with DTC-2 suffer a higher relative risk of cardiovascular death compared to the general population. In conclusion, clinicians should be more careful to administer THST for younger DTC-2 patients but more audacious for the elder ones.

Cancer treatment, whether through radiation or chemotherapy, introduces additional layers of cardiovascular risk [41]. It is not implausible that radiotherapy, especially thoracic radiotherapy [42], increases the risks of CVD [41, 43] and cardiovascular mortality [44-46] in cancer survivors involving thyroid cancer. In addition, chemotherapy such as anthracycline may harm the circulatory system through oxidative stress reactive and oxygen species [47, 48]. Our data indicates that DTC-2 patients, especially those previously treated with radiotherapy or chemotherapy for their initial cancer, are at an increased risk of cardiovascular mortality. Our analysis in DTC patients with thoracic cancers as their first malignancies further reflected that the cardiac toxicity caused by thoracic radiotherapy may attribute to CVD. This is less attributable to treatment for DTC, given the lower incidence of intensive treatment (eg, radiotherapy or chemotherapy) in DTC-2 compared to DTC-1 patients. However, we also observed increased risk among DTC-2 patients who did not receive chemotherapy or radiotherapy for their first neoplasm. This implies that the observed increase in cardiovascular mortality risk among DTC-2 patients persists irrespective of “extra” cancer treatment for their initial malignancy, indicating that the elevated risk cannot be solely explained by the additional cancer treatment necessitated by the first malignancy.

Lifestyle factors, such as smoking and alcohol consumption, may predispose individuals to both DTC-2 and CVD [6, 7], a correlation evident in our subgroup analysis of patients with a prior history of laryngeal cancer—a group predominated by drinkers and smokers [49]. We also found a similar correlation in a larger subgroup of smoking-related cancers as their first malignancies. Hence, is there a possibility that a DTC-2 is an accidental event in a patient who has shared risks for both neoplasms and CVD? Nevertheless, the case-crossover study design supports the assertion that the association between DTC-2 and cardiovascular mortality extends beyond these shared risk factors. This analysis also balances unmeasured risk factors for CVD (eg, hyperlipemia and diabetes) that are supposedly stable in a short period.

It has been proven that psychological stress may prompt cardiovascular mortality [50], especially in a vulnerable cohort. Stress is implicated in heightened risk for cardiac regulatory alterations [51], potentially via the sympathetic nervous system and hemostatic activation [52, 53]. The experience of being diagnosed as and living with cancer imposes significant stress. Substantial evidence has demonstrated that risks for CVD or cardiovascular mortality [54], mental health disorders [55], and suicide [56, 57] increase sharply after an initial malignancy diagnosis. For instance, data suggests that the risk of cardiovascular mortality within the first month proceeding a cancer diagnosis is doubled compared to individuals without cancer [19], underlying the acute and significant impact of stress on cardiovascular health induced by such a diagnosis. Yet, the response to a secondary malignancy remains underexplored. Echoing the literature pertaining to first malignancies, our research indicates that the risk of cardiovascular mortality is far more markedly elevated within the first month following a diagnosis of DTC-2 compared with the general population. This heightened risk may also be attributable to cancer-induced alterations in coagulation, potentially leading to arterial thrombosis through tumor cell interactions with, and activation of, the host's hemostatic mechanisms [58, 59]. Notably, studies such as those by Navi et al [58] and Gross et al [59] have identified an increased incidence of myocardial infarction and in-stent thrombosis, respectively, in patients recently diagnosed with cancer, further substantiating the interrelation between cancer diagnosis and adverse cardiovascular events.

The principal strength of our investigation is its population-based prospective cohort design, which minimizes recall and selection biases. The substantial cohort size enables a comprehensive analysis of interactions with variables such as attained age, time since diagnosis, and treatment modes. A primary concern is the potential misclassification of causes of death. Nonetheless, the likelihood of differential misclassification between patients diagnosed as DTC and the general population is small. Second, certain factors associated with CVD or survival, such as tobacco use, comorbidities, and treatment modality including checkpoint inhibitors and THST (eg, the dose of levothyroxine or the level of TSH), were not recorded in our study, nor were the details of radioactive iodine treatment such as the total number of ^131^I treatment for each patient. However, we adopted some alternative factors (ie, suffering a smoking-related cancer or thoracic cancer as the first malignancy) in subgroup analysis and conducted a nested case-crossover analysis to partially eliminate the confounding bias. Third, the inclusion of individuals with cancer in the aggregated population data may underestimate the true associations. Fourth, there is a possibility that metastatic events from the primary malignancy could be misclassified as DTC-2. However, given the stringent pathological diagnostic criteria for inclusion and the SEER Program's policy of classifying ambiguous cases as metastases [60], it is less likely that such misclassification may influence the results. Additionally, we have also observed elevated risk in DTC-2 patients with a localized first malignancy. It is also important to consider the role of competing risks, such as the primary cancer, which may prevent patients from dying of CVD. Reassuringly, in this case, an underestimated cardiovascular mortality risk among DTC-2 patients would have been anticipated. Moreover, there is a demographic distinction between DTC-2 and DTC-1 patients, particularly in age and follow-up duration (attributable to different overall survival). However, our analyses have consistently been stratified by attained age groups and time since cancer diagnosis, ensuring comparability between groups.

Our study also lacked information on radiotherapy sites. The association demonstrated that patients who underwent radiotherapy for their previous malignancy may be confounded with treatments targeting sites other than the chest, which are less likely to increase cardiovascular mortality risk. Consequently, any associated increase in risk is likely undervalued. Last, to ensure sufficient sample size, individuals without information on previous malignancy were not excluded, which might enroll patients with a prior history of DTC in the baseline cohort. However, considering patients with a DTC history account for a tiny part of DTC-2 patients, the analysis results should not be affected.

## Conclusions

Our study indicates an increased cardiovascular mortality risk in DTC-2 patients compared with their DTC-1 counterparts and the general population. Though DTC-2 may be considered an uncontrollable risk factor for cardiovascular mortality, our findings emphasize the importance of vigilant cardiovascular monitoring and cautious THST in DTC-2 patients, especially for those under 75 years old. Notably, the pronounced risk observed during the first month following a DTC-2 diagnosis reminds us to integrate stress management into the cancer care protocol.

## Data Availability

Original data generated and analyzed during this study are included in this published article or in the data repositories listed in References.

## Abbreviations

CI: confidence interval
CVD: cardiovascular disease
DTC: differentiated thyroid cancer
DTC-1: differentiated thyroid cancer as a first malignancy
DTC-2: differentiated thyroid cancer as a second malignancy
IRR: incidence rate ratio
OR: odds ratio
SEER: Surveillance, Epidemiology, and End Results
THST: thyroid hormone suppression therapy
